# Editorial for Delayed Perforation after Colorectal Cold Snare Polypectomy with Simultaneously Performed Endoscopic Submucosal Dissection: A Case Report and Literature Review

**DOI:** 10.31662/jmaj.2025-0262

**Published:** 2025-06-20

**Authors:** Masaaki Kodama

**Affiliations:** 1 Department of Advanced Medical Sciences, Oita University, Yufu, Japan; 2 Department of Gastroenterology, Oita University, Yufu, Japan

**Keywords:** colorectal tumor, cold snare polypectomy, perforation, bleeding

Colorectal cancer has been increasing in incidence and mortality in Japan and worldwide in recent years. While *Helicobacter pylori* is known to be a major pathogen of gastric cancer, various risk factors such as lifestyle, diet, lack of exercise, obesity, smoking, alcohol consumption, and other factors are involved in the risk of colorectal cancer. Therefore, elucidation of carcinogenesis mechanisms and development of more detailed, efficient, and safe diagnostic and therapeutic methods for colorectal cancer are needed.

Endoscopic screening is important for the early detection of colorectal cancer. Endoscopic resection of neoplastic lesions in the colon and rectum has been shown to reduce the incidence of colorectal cancer. Moreover, endoscopic resection of colorectal neoplasm, such as adenomas, prevents death from colorectal cancer. Adenoma-carcinoma sequences are associated with the development of colorectal cancer, and appropriate management of adenoma by endoscopy is very important.

In recent years, cold snare polypectomy (CSP) has become a widely adopted method worldwide for endoscopic polyp resection of the colon due to its usefulness, simplicity, and safety. CSP is a technique for resecting colorectal tumors without the use of electrocautery.

The Japanese Society of Gastrointestinal Endoscopy (JSGE) published the second edition of the “Guidelines for Colorectal Endoscopic Submucosal Dissection/Endoscopic Mucosal Resection” in 2019. In addition, JSGE supplementally published a guideline for CSP in 2022 ^(1)^.

In this guideline, as the recommendation for the clinical question of CSP indication, the following is stated: ‘The indications should be limited to lesions smaller than 10 mm that are preoperatively diagnosed as adenoma and which can be resected completely en bloc.’ (Strength of recommendation: 1, Evidence level: B) ^(1)^.

Many randomized controlled trials (RCTs) and meta-analyses have been reported on the comparison between CSP and hot polypectomy. Recently, O’Sullivan et al. ^(2)^ reported results from an RCT comparing CSP and hot snare polypectomy (HSP) in flat, non-pedunculated colorectal polyps larger than 15 mm. “¥HOT” refers to the technique using electrocautery. In this study, it is reported that delayed perforation was seen only in one case of HSP but not in CSP, bleeding was more common in HSP, and recurrence was significantly more common in CSP. This result suggests that CSP is a very safe treatment technique, but there is a limitation regarding recurrence.

Takeuchi et al. ^(3)^ reported in their review article that CSP is indicated for adenomas less than 10 mm because of its safety and simplicity, with little delayed bleeding. However, for polyps larger than 10 mm, CSP is a difficult candidate and requires further close examination ^(3)^. In 2025, Sorge reported in their meta-analysis that CSP showed double the recurrence risk of hot endoscopic mucosal resection (H-EMR) for nonpedunculated colorectal polyps, however, there were no intraprocedural or delayed perforations in the CSP group ^(4)^. Although CSP has a higher recurrence rate than H-EMR, its superior safety makes it highly useful, even in cases with extensive comorbidities.

In the CSP guideline’s statement, it is revealed, “It is likely that postprocedural bleeding occurs less frequently and perforation is encountered negligibly in cold polypectomy compared with endoscopic resection with electrocautery”. (Strength of recommendation: None, Evidence level: C) ^(1)^.

In addition, the guideline also stated that “It is likely that the risk for postprocedural bleeding in patients on antithrombotic therapy is lower with cold polypectomy than with endoscopic resection with electrocautery”. (Strength of recommendation: None, Evidence level: C) ^(1)^.

In the present case report, Matsumoto et al. ^(5)^ reported a case of delayed perforation after CSP (although endoscopic submucosal dissection (ESD) of other sites was performed simultaneously), along with a literature review. The authors also discussed patient background factors and comorbidities, especially oral steroids and chronic kidney disease (CKD) as risk factors for delayed perforation with CSP. In this case report, the authors described that the influence of comorbid CKD was thought to be indicated.

This case is very interesting as an example of delayed perforation associated with CSP and provides valuable and informative suggestions for the worldwide practice of CSP. Limitations of this report include the lack of specific background factors such as steroid use and CKD in this case, and the influence of ESD performed at other sites, However, a detailed search of other articles also analyzed the risk of perforation. This review also indicates very useful suggestions for safer endoscopic treatment for colorectal neoplasms.

The Japan gastrointestinal endoscopy society reported the national survey of adverse events in gastrointestinal endoscopy. This report revealed that adverse event rates were 0.076% in observation-only endoscopy and 1.145% in therapeutic endoscopy, respectively. Most serious adverse events occurred in elderly patients. In both observational and therapeutic endoscopy, it is important to pay close attention to the many background risk factors in patients.

CSP is expected to be performed more frequently in Japan and worldwide, where the number of colorectal cancer patients is increasing. Careful application of CSP with careful attention to background factors is recommended. Further studies are needed to clarify the safety and utility of the CSP method and its indication to prevent colorectal cancer development.

## Article Information

### Conflicts of Interest

None

### Author Contributions

The authors alone are responsible for the content and writing of the paper.
